# Berberine Ameliorates DSS-Induced Colitis via Regulation of Mucosal Barrier Homeostasis and Mucin-Degrading Microbiota

**DOI:** 10.3390/ijms27031549

**Published:** 2026-02-04

**Authors:** Yanli Chen, Yan Wang, Yanmin He, Lei Qiao, Weilong Dai, Yalin Liu, Xiaoxi Lu, Yujie Gan, Lu Sun, Mingzhi Yang, Yizhen Wang, Jie Fu, Mingliang Jin

**Affiliations:** 1 Institute of Feed Science, College of Animal Sciences, Zhejiang University, Hangzhou 310058, China; 2 Key Laboratory of Molecular Animal Nutrition, Ministry of Education, Zhejiang University, Hangzhou 310058, China; 3 Key Laboratory of Animal Nutrition and Feed Science (Eastern of China), Ministry of Agriculture, Hangzhou 310058, China; 4 National Engineering Research Center for Green Feed and Healthy Breeding, Hangzhou 310058, China; 5 Zhejiang Key Laboratory of Nutrition and Breeding for High-Quality Animal Products, Hangzhou 310058, China

**Keywords:** berberine, gut microbes, mucin, SCFAs, intestinal barrier

## Abstract

Berberine, a benzyl isoquinoline alkaloid, is used in food for its diverse spectrum of biological activities. Inflammatory bowel disease (IBD) is a widespread condition characterized by frequent occurrence and limited therapeutic success. Berberine has been shown to alleviate colitis through enhancement of the intestinal barrier and modulation of gut microbial balance. However, the further mutualistic balance mechanism between microbes and the mucus of berberine in alleviating IBD still needs to be clarified. Our findings demonstrated a strong association between berberine’s therapeutic efficacy and alterations in the gut microbiota. This includes enhancements in the level of IgA-coated bacteria, Zg16, Reg3g, and Pla2g2a, all of which contribute to microbiota homeostasis. Moreover, the beneficial effect on gut barrier function of berberine was mostly attributed to *Akkermansiam* and *Bacteriodes*-associated mucin–SCFA metabolism. This study lays a critical groundwork for the development of berberine-based functional food additives that harness its nutraceutical potential.

## 1. Introduction

A diverse range of herbal feed additives is marketed to meet the growing demand from livestock producers for enhancing animal health and production performance. Among these supplements, berberine-containing products are particularly widespread [1]. Berberine, a benzyl isoquinoline alkaloid, can be extracted from the roots of Rhizoma coptidis and the berries of plants such as Berberis vulgaris and Coptis chinensis [1,2]. As a plant-derived nutraceutical compound, berberine has attracted considerable interest for its health-promoting and therapeutic properties [3]. The compound has been a food additive for exhibiting a diverse spectrum of biological activities, including anti-diabetic, hypolipidemic, antimicrobial, antiviral, anticancer, anti-obesity, anti-inflammatory, and antidepressant effects [4,5,6]. Inflammatory bowel disease (IBD) in livestock is a chronic condition marked by persistent inflammation of the gastrointestinal tract, primarily driven by elevated levels of pro-inflammatory cytokines and compromised epithelial barrier disorder [7,8]. The increasing prevalence of IBD in the production of animals has been linked to intensive farming practices and changes in dietary formulations [9]. Recent studies have highlighted berberine as a promising agent for alleviating gut inflammation and improving gut mucosal barrier function [1,10].

The mucus layer comprises two distinct components, with the inner layer tightly adhering to the epithelium. This inner layer is typically devoid of bacteria and enriched with protective antibacterial peptides such as defensins and lysozyme [11,12]. The outer layer, in contrast, is thicker and loosely adherent, serving as a habitat rich in commensal microbes and their metabolic products [13]. The main structural components of mucus are mucins, primarily secreted by goblet cells, which play a crucial role in protecting the intestines from mechanical, chemical, and microbiota-related challenges [14]. Additionally, they help maintain the integrity of vulnerable areas in the body, including the intestinal mucosal and epithelial barriers [15]. The biosynthesis and secretion of mucins is a complex process, with Mucin 2 (MUC2) serving as the primary glycoprotein in intestinal mucus. MUC2 contains both O- and N-linked glycans, and its glycosylation takes place in the endoplasmic reticulum (ER) and Golgi apparatus, involving the co-ordinated action of multiple glycosyltransferases (e.g., sialyltransferases, fucosyltransferase) [16]. In livestock, abnormal MUC2 synthesis and secretion disrupt host–microbial interactions, leading to intestinal inflammation and a compromised mucin barrier. This impairment increases the risk of poor nutrient digestion and absorption, ultimately reducing animal productivity [17,18].

Mucins are also essential for facilitating the colonization of gut microbiota within the gastrointestinal tract and for preserving the intricate equilibrium between the host and its microbial community [19,20]. Gut microbes can exploit mucin-derived glycans as a nutritional substrate, and numerous gut bacteria demonstrate accelerated growth when cultured in mucin-enriched media. Notably, the mucin-degrading bacterium *Akkermansia* spp. and *Bacteriodes* spp. thrives under these conditions [21,22]. Mucolytic bacteria modulate host–microbiota symbiosis through their ability to degrade mucin glycans to produce short-chain fatty acids (SCFAs) [23]. In animal production, enteric disorders often compromise the host–microbiota mutualism—a key relationship essential for intestinal homeostasis. Recent research indicates that the phytogenic compound berberine, utilized as a dietary supplement, can effectively improve gut mucosal barrier integrity and re-establish microbial homeostasis in experimentally induced colitis models [24]. However, the mechanism by which berberine mediates the microbiota-mucus interaction to ameliorate inflammatory gut conditions in animals still requires clarification. This work aimed to address this gap and explore the underlying pathways in order to establish a mechanistic basis for its development and rational use as a nutraceutical in animal production.

## 2. Results

### 2.1. Berberine Relieves Mouse Colitis Symptoms Induced by DSS

**Figure 1:**
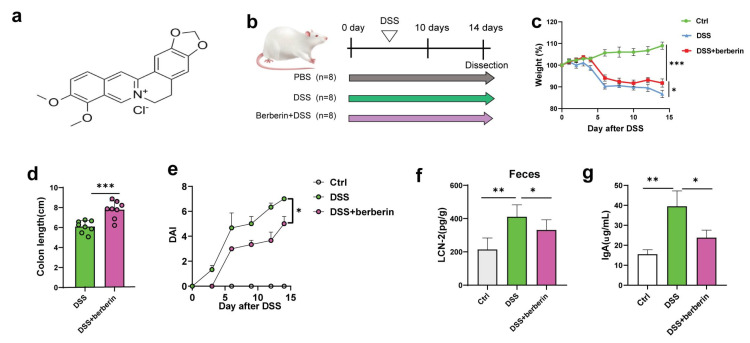
Berberine relieved DSS-induced colitis in mice. (**a**) Chemical structure of berberine. (**b**) Schematic diagram of the experiment. Arrows of different colors represent different groups. (**c**) Body weight percentage changes. (**d**) Colonic length. (**e**) The effect of berberine on DAI in mice. (**f**) LCN-2 levels in the feces. (**g**) IgA levels in the feces. * *p* < 0.05, ** *p* < 0.01, *** *p* < 0.001.

The structure of berberine is depicted in Figure 1a. In this study, acute colitis was successfully induced in C57BL/6 mice by administering 2.5% DSS in drinking water for 10 consecutive days according to a previous study (Figure 1b) [25]. This model exhibited phenotypic characteristics comparable to those observed in human IBD. During model induction, mice in the DSS group experienced notable weight loss. However, berberine administration significantly alleviated DSS-induced colitis, as evidenced by reduced weight loss compared to the untreated group (Figure 1c). Colon shortening is a hallmark feature of DSS-induced colitis, and treatment with berberine markedly attenuated this pathological alteration (Figure 1d). Similarly, berberine significantly lowered the disease activity index (DAI) score, an established indicator of colitis severity in experimental models (Figure 1e). The DAI was markedly elevated in the DSS group. Berberine treatment significantly ameliorated these symptoms, consistent with findings reported in previous studies [25]. In addition, as the major biomarkers for colonic inflammation [26,27], the level of fecal LCN-2 and IgA was decreased (Figure 1f,g). These results suggest a potentially important role of berberine in alleviating IBD and promoting intestinal homeostasis.

### 2.2. Berberine Relieves Colonic Intestinal Barrier Dysfunction

**Figure 2:**
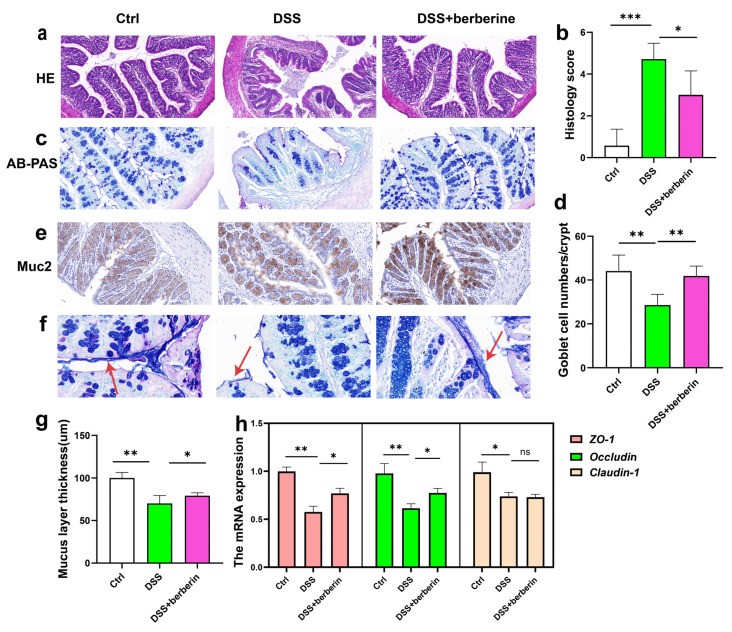
Berberine relieved DSS-induced gut barrier integrity in mice. (**a**) H&E staining sections of colon (magnification of 40×). (**b**) Histological scores of colon tissue. (**c**) AB-PAS staining sections of colon (magnification of 40×). (**d**) The number of goblet cells/crypt. (**e**) Representative images of Muc-2 protein expression in the colonic tissues (magnification of 60×). (**f**,**g**) Representative images of the thinness of the mucus layer (magnification of 100×). The red arrow points to the mucus layer. (**h**) The relative mRNA expression level of the tight junction protein (Zo-1, Occludin, Claudin-1). * *p* < 0.05, ** *p* < 0.01, *** *p* < 0.001.

Tissue specimens from the colon were collected and processed for histological evaluation. Observations of the proximal colon revealed that berberine effectively mitigated intestinal barrier disruption induced by DSS (Figure 2a,b). To further evaluate the integrity of the intestinal mucosal barrier following berberine treatment, colonic tissues were subjected to AB-PAS staining, which selectively highlights glycosylated mucin proteins produced by goblet cells (Figure 2c,d). As shown in Figure 2c, the loss of goblet cells in gut epithelium and distorted crypts was found in the DSS group; berberine treatment led to significant histological improvements, including notable enhancements in crypt architecture, an increased number of goblet cells (Figure 2d), decreased mucosal injury, and increased mucus layer thickness (Figure 2f,g). MUC2 is mainly secreted from goblet cells and is the main mucus component in the colon, which contributes to forming the natural barrier between the host and gut microbes. Immunohistochemical analysis of MUC2 expression showed a marked increase in MUC2 levels in the DSS + berberine group compared to the DSS group (Figure 2e). Furthermore, our findings indicate that the therapeutic effect of berberine on colitis is associated with the upregulation of the mRNA expression of tight junction proteins, including Zo-1 and Occludin, thereby enhancing gut epithelial barrier integrity (Figure 2h). In summary, berberine alleviates intestinal permeability and enhances the expression of proteins associated with mucosal homeostasis, thereby mitigating intestinal inflammation.

### 2.3. Berberine Relieves Colon Mucus Production and Secretion

**Figure 3:**
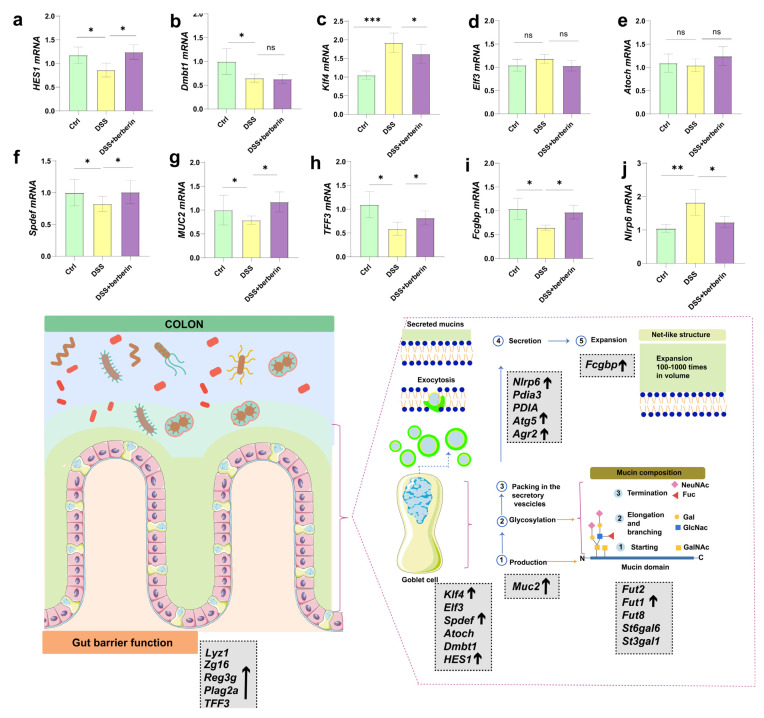
Berberine impacts mucus production and secretion. (**a**–**g**) Relative mRNA expression levels of transcription factors involved in goblet cell differentiation in colonic tissues: (**a**) Hes family basic helix-loop- helix (bHLH) transcription factor 1 (*Hes1*), (**b**) deleted in malignant brain tumor protein1 (*Dmbt1*), (**c**) Kruppel-like factor 4 (*Klf4*), (**d**) E74-like ETS transcription factor 3 (*Elf3*), (**e**) atonal BHLH transcription factor (*Atoch*), (**f**) SAM pointed domain containing ETS transcription factor (*Spdef*), and (**g**) Muc2, (**h**) Trefoil factor 3 (Tff3), (**i**) IgGFc-binding protein (Fcgbp), and (**j**) NOD-like receptor family pyrin domain containing 6 (*Nlrp6*). The black arrows represent upregulation of the gene. * *p* < 0.05, ** *p* < 0.01, *** *p* < 0.001, ns *p* > 0.05.

We next investigated whether the beneficial effects of berberine on gut barrier function were associated with alterations in intestinal mucus production and secretion. Our results demonstrated that berberine significantly modulated the dysregulated expression of genes associated with goblet cell differentiation. Specifically, *Hes1* and *Spdef* expression levels were elevated, while *Klf4* expression was reduced in the colons of DSS + berberine mice compared to those receiving DSS alone (Figure 3a–f). Mucus contains a number of other molecules. Specific mucus components, such as FCGBP and TFF3, are produced together with MUC2 and secreted by goblet cells and are integrated into the mucus to maintain the structure of the mucus layer (Figure 3g–i). These critical mucus components were upregulated after berberine administration. We next observed that berberine treatment selectively modulated the expression of mRNA associated with intestinal mucus secretion (Figure S1a–j). In particular, berberine supplementation showed a trend toward increased expression of *Atg5* (Figure S1c) while decreasing another key marker (*Nlrp6*) in the colon (Figure 3j). The results indicate a potential balance in mucus production following oral administration of berberine. Finally, we set out to assess whether berberine treatment impacts intestinal mucin glycosylation; we measured the expression of glycosyltransferases (Figure S1f–j). We found some genes of enzymes, such as fucosyltransferase (Fut1, Fut2) (Figure S1i,j) and sialyltransferase (St3gal1, St6gal6) (Figure S1f,g), were disordered in the DSS group compared with healthy mice. Among the genes of glycosyltransferases, Fut1 showed a downregulation tendency after berberine administration. Our data showed that berberine could regulate the disorder of mucin induced by DSS.

### 2.4. The Level of IgA-Coated Bacteria and Defensins Was Reversed by Berberine

**Figure 4:**
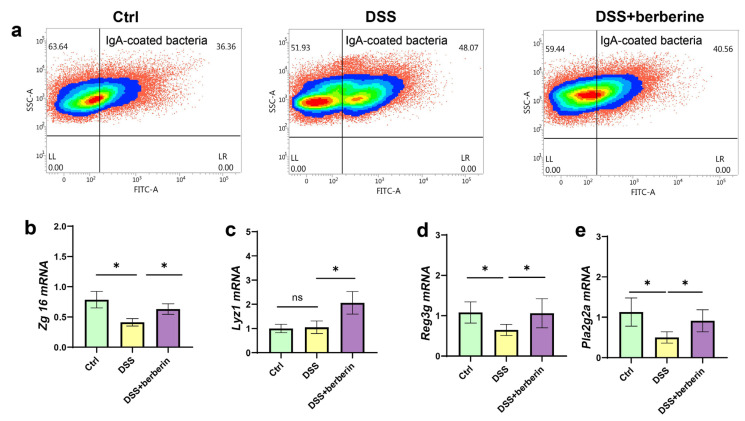
Berberine reversed the DSS-induced reduction in IgA-coated bacteria and defensins. (**a**) Percentages of total IgA-coated bacteria in the feces by flow cytometer; IgA-coated bacteria were labeled with FITC. The color in a cell density plot represents the number of cells. (**b**–**d**) Antimicrobial peptides mRNA expression: (**b**) Zymogen Granule Protein 16 (Zg16), (**c**) lysozyme C (Lyz1), (**d**) regenerating islet-derived 3-gamma (Reg3g), and (**e**) phospholipase A2 group II (Pla2g2a). * *p* < 0.05, ^ns^ *p* > 0.05.

To the best of our knowledge, mucins and their associated components play a crucial role in maintaining spatial separation between gut microbiota and the intestinal epithelium [19]. The impaired mucus layer led to bacteria penetrating the epithelium, triggering host immune response and aggravating colitis. However, the host can also secrete antibacterial agents in mucus, such as defensins and IgA, to interact with gut microbes directly and maintain host–microbiota hemostasis [11,28]. Thus, we detected the level of IgA-coated bacteria. Consistent with the previous report, IgA production, a common anti-microbial response in the gut, was also usually upregulated during colitis in our study [29], and the concentration was reduced by berberine treatment (Figure 1f). In addition, the proportion of lgA-coated bacteria was higher in the DSS group than in the DSS + berberine group (Figure 4a). The levels of Zg16, Reg3g, and Pla2g2a were reduced, whereas Lyz1 expression remained relatively unchanged after DSS treatment. Berberine treatment further significantly upregulated the mRNA expression levels of Zg16, Lyz1, Reg3g, and Pla2g2a (Figure 4b–e). These findings suggest that berberine treatment maintains microbial homeostasis by modulating the immune response associated with gut microbiota.

### 2.5. Berberine Changed the Gut Microbiota Perpetuation Induced by DSS and Increased the Akkermansia and Bacteroides Level

**Figure 5:**
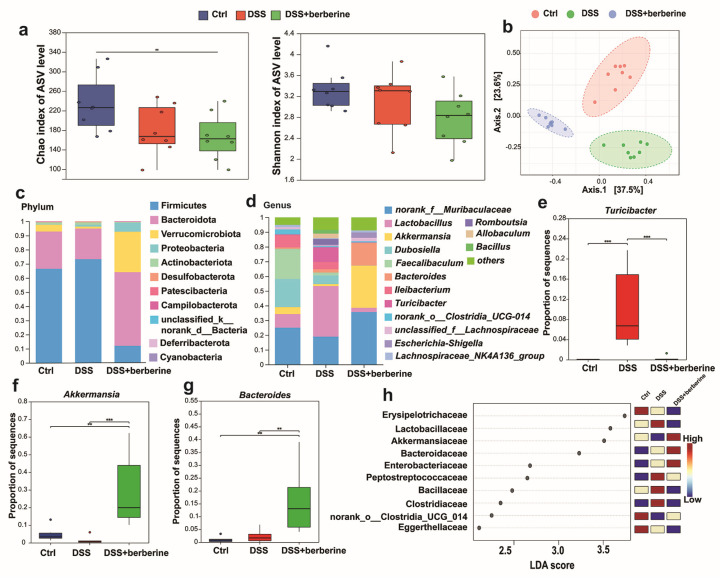
Berberine changes the gut microbiota perpetuation induced by DSS. (**a**) Microbial α-diversity (Chao and Shannon indices). (**b**) Principal co-ordinate analysis (PcoA) at the ASV level for samples from colonic contents based on the Bray–Curtis distance. (**c**,**d**) Bacterial taxonomic profiling at the phylum and genus levels of intestinal bacteria resulting from different groups. The relative abundance of bacteria, including *Turibacter* (**e**), *Akkermansia* (**f**), and *Bacteroides* (**g**) were different between three groups. (**h**) The biomarkers among different groups according to LEfSe analysis. * *p <* 0.05, ** *p <* 0.01, *** *p <* 0.001.

We performed 16S rRNA sequencing to further analyze the specific regulatory role of berberine on the gut microbiota. The result showed that the Chao indices was reduced in the DSS-induced colitis group and the DSS + berberine group compared to the control group. However, there are no significant differences in the Shannon index among the three groups (Figure 5a). PCoA analysis revealed distinct clustering of gut microbiota compositions among the different experimental groups, indicating clear group-specific microbial community structures (Figure 5b). At the phylum level, the *Firmicutes*/*Bacteroidota* ratio was increased in the DSS group, and berberine treatment significantly reversed this trend by reducing the abundance of *Firmicutes* and lowering the *Firmicutes*/*Bacteroidota* ratio (Figure 5c). In addition, we analyzed the compositional differences in gut microbiota at the genus level (Figure 5d). The relative abundance of the pathogenic genus *Turicibacter* was significantly elevated in the DSS group compared to both the control and DSS + berberine groups (Figure 5e), whereas mucin-utilizing bacteria (*Akkermansia* and *Bacteroides*) were noticeably increased in the DSS + berberine group (Figure 5f). Furthermore, *Akkermansia* and *Bacteroides* were the significant biomarkers in the DSS + berberine group based on LEfSe analysis (Figure 5h).

### 2.6. Berberine Changes the Mucin–Microbe Mutualistic Relationship Induced by DSS

**Figure 6:**
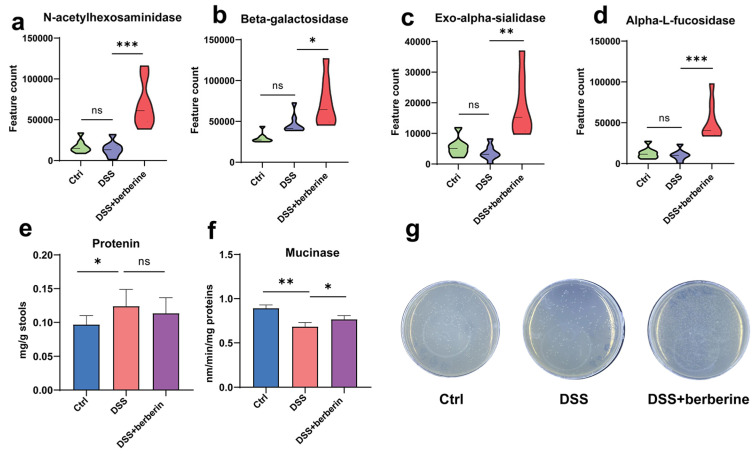
Effects of berberine supplementation on the microbe–mucin axis. (**a**–**d**) The microbiota enzymes capable of hydrolyzing mucin based on PICRUSt2. (**e**) Protein content and (**f**) mucinase activity for porcine stomach mucin were detected in fecal supernatant. (**g**) Bacterial plating with porcine stomach mucin as a carbon source after culturing for 24 h. * *p* < 0.05, ** *p* < 0.01, *** *p* < 0.001, ns *p* > 0.05.

Given the change in mRNA expression of *MUC2* and glycosyltransferases in colon tissue in our previous study, we hypothesized that the quantities of mucin and mucin glycan may be affected. Mucin glycans form core structures, consisting of N-acetylgalactosamine, galactose, and N-acetylglucosamine, fucose, sialic acids, and sulfate residues in the colon, which can be utilized by gut microbes to contribute to microbiota balance in healthy mice. To verify whether berberine modulates gut microbiota to alter the fecal mucin ability of feces, we conducted function prediction analysis according to PICRUSt2. We discovered that some glycoside hydrolase enzymes capable of hydrolyzing mucin were significantly more upregulated in the DSS + berberine group than in the DSS group. However, these enzymes were similar between the Ctrl and DSS groups (Figure 6a–d). Therefore, we speculated that microbiota mucinase activity was increased, and we conducted further verification in vitro. Notably, the mucinase of fecal supernatant was higher in DSS + berberine mice than in Ctrl and DSS groups, which was consistent with 16S prediction about mucinase activity analysis (Figure 6e,f). Furthermore, berberine treatment resulted in a greater increment of bacteria with mucin-utilizing ability within feces compared to the DSS group when fecal microbes were cultured using mucin as the sole carbon source (Figure 6g). These findings showed that there are more bacteria that utilize mucin as their carbon source to survive, demonstrating that beneficial *Akkermansia* and *Bacteroides* can utilize mucin in the gut to promote colitis in mice.

### 2.7. Modulation of the Gut Microbiota Contributed to the Improvement of Gut Barrier Function by Increasing SCFAs Production Through Mucin Utilization During Berberine Treatment

**Figure 7:**
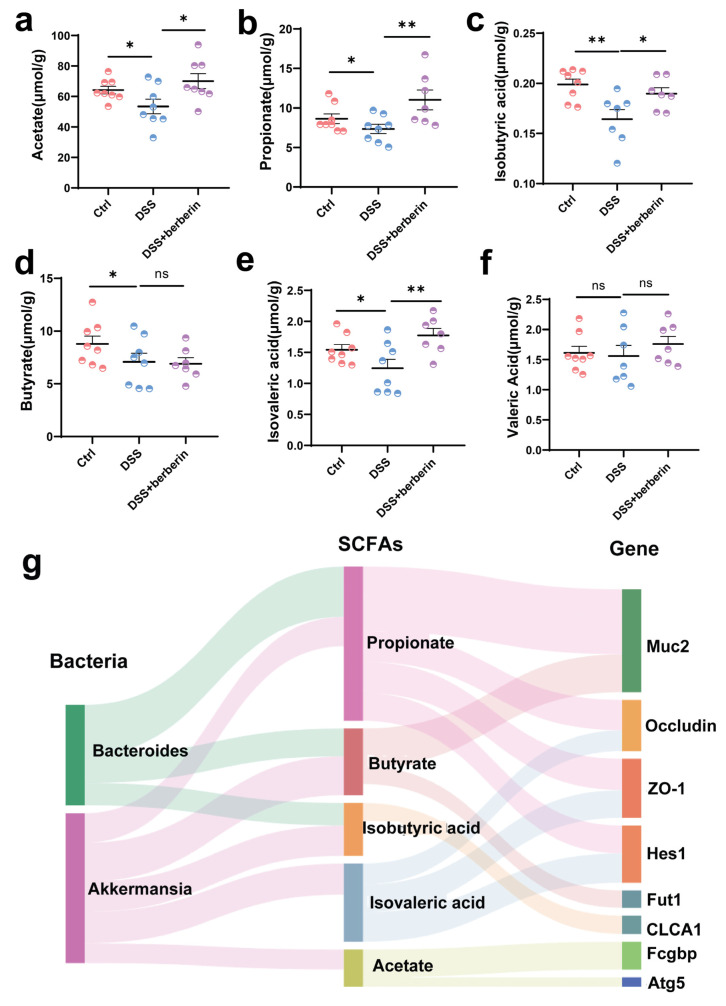
Effects of berberine supplementation on the mucin–SCFA axis. (**a**–**f**) SCFAs production by gut microbiota in the feces. (**g**) A dynamic interactive multilayer Sankey diagram based on significant genus (Spearman’s r > 0.24, *p <* 0.05). * *p* < 0.05, ** *p* < 0.01, ns *p* *>* 0.05.

To directly assess whether gut microbiota could utilize fecal mucin as a fermentation substrate to produce SCFAs, our results showed that berberine supplementation significantly increased the levels of most SCFAs, with the exception of valeric acid (Figure 7a–f). Thus, microbiota utilization for mucin glycan may be a key determinant for the maintenance of SCFAs production in our model. Our correlation analysis accounted for the improvement of gut barrier through the SCFAs triggered by an increase in the relative abundance of mucin-utilizing bacteria after berberine treatment (Figure 7g).

## 3. Discussion

IBD-related intestinal disorders affect a significant population of livestock globally, with incidence rising steadily across various intensive production systems. These conditions pose substantial economic losses and animal welfare concerns [30,31]. Nutraceutical berberine has shown significant therapeutic potential against DSS-induced gut mucosal disorder depending on gut microbiota [24,32]. However, the therapeutic mechanism, through regulating the microbiota–mucosal mutualistic correlation to restore the intestinal barrier function, remains incompletely understood. Our study was in agreement with previous studies [25,33,34]; berberine significantly improved intestinal barrier integrity, as well as alleviated colitis-related symptoms. We confirmed that berberine treatment effectively suppressed hematochezia, reduced the DAI, promoted restoration of colon length, and lowered fecal LCN-2 levels compared to mice treated with DSS alone. In addition, berberine significantly preserved the loss of mucosal integrity in DSS mice.

To further investigate the mechanisms underlying gut barrier regulation, we focused on mucin. In vivo studies have shown that berberine enhances Muc2 expression in mice under inflammatory conditions [35,36]. However, there were no studies to investigate the effect of berberine supplementation on the synthesis, glycosylation, and secretion of mucin. Our research demonstrated that berberine supplementation reversed the DSS-induced alterations in the expression of several key markers involved in goblet cell differentiation (e.g., *Klf4*, *Hes1*, and *Spdef*) as well as in the synthesis and secretion of major mucous layer components (e.g., TFF3 and MUC2). In addition, berberine treatment reversed the expression of several markers associated with mucus secretion and stabilization (e.g., *Atg5* and *Nlrp6*) and upregulated Fut1, a key gene involved in mucus glycosylation. These effects were associated with an increased accumulation of mucin in the colons of berberine-treated mice, suggesting that berberine enhances epithelial protection by limiting bacterial translocation across the mucosal barrier.

Dysbiosis of the gut microbiota compromises mucosal barrier integrity and aggravates intestinal inflammation [37,38]. Defensins, endogenous antimicrobial peptides produced by Paneth cells and other epithelial cells [39,40], are key components of the mucus layer with potent microbicidal activity against pathogenic bacteria [41]. Disturbed antimicrobial agents seem to be a critical factor in inflammatory disease of the intestinal tract [42]. Can berberine regulate defensin expression to alleviate changes in gut microbiota and colitis? We discovered that berberine could regulate gut microbiota perpetuation by increasing the expression level of Lyz1, Zg16, Reg3g, and Pla2g2a. Notably, REG3G is a potential mediator of host–microbiota interactions and has been shown to exert multiple beneficial effects on intestinal function and homeostasis [43,44]. In addition, IgA is the predominant antibody isotype produced at mucosal surfaces and is a critical mediator of intestinal health and microbiota homeostasis [45]. IgA-coating bacteria was higher in inflammatory bowel disease than healthy individuals [46], which was similar to our study. The results implied that berberine could improve colitis by increasing defensins and IgA-coating bacteria to change the gut microbiota community.

Although existing research has suggested that berberine can reverse the disorder of microbiota [47], the α-diversity of gut microbiota in the DSS + berberine group was similar to that of the DSS group. However, PCoA analysis demonstrated that the microbiota were totally distinguished from each other. These results showed that gut microbiota changed after berberine administration compared with the Ctrl group, rather than fully restoring the gut microbial community. It was reported that gut microbes could utilize mucin as a carbon source [48,49].

Further microbial function analysis revealed that mucin utilization capacity was significantly increased in the DSS + berberine group. However, the absence of a difference between the Ctrl and DSS groups could be due to the lack of changes in certain gut microbiota. Certain gut bacteria, such as *Akkermansia*, are known to utilize mucin-derived glycans rather than dietary polysaccharides as their primary carbon source. Notably, *Akkermansia* emerged as a predominant genus in the DSS + berberine group. *Akkermansia* [50,51,52] has been reported to alleviate mucosal inflammation and modulate microbial composition in mice with DSS-induced colitis. *Bacteroides*, SCFA producers [53,54], were significantly upregulated in DSS + berberine-treated mice. *Bacteroides* were obviously fewer in IBD patients than in healthy individuals. We hypothesize that mucin glycans can be fermented by the gut microbiota to produce SCFAs, a mechanism that supports intestinal barrier integrity in livestock. Furthermore, correlation analysis between SCFAs production with beneficial microbes like *Akkermansia* and *Bacteroides*, suggesting their functional role in reinforcing the mucus layer as part of a host–microbe symbiosis crucial for gut health.

## 4. Materials and Methods

### 4.1. Experimental Animals

Eight-week-old male C57BL/6 mice (Ctrl: *n* = 8; DSS: *n* = 8; DSS + berberine: *n* = 8) were used in this study. All animals were obtained from the Laboratory Animal Center of Zhejiang University and acclimated for one week prior to the start of experiments. Mice were housed in a specific pathogen-free facility within the breeding room of the same center. Throughout the study, animals were maintained under standardized conditions with ad libitum access to food and water. The animal experiment was approved by the Laboratory Animal Welfare and Ethics Review Committee of Zhejiang University (Approval No. ZJU20250961) and conducted in accordance with ethical standards.

### 4.2. Establishment of DSS Model and Administration of Berberine

Mice were given ad libitum access to water containing either 2.5% Dextran Sulfate Sodium (DSS) (MeilunBio, 9011-18-1, Dalian, China) or water for 10 days. This was followed by daily oral administration of 200 μL PBS, with or without berberine at a dose of 100 mg/kg body weight, for an additional 7 days. Berberine (purity ≥ 98%) was obtained from company (Shanghai yuanye Bio-Technology Co., Ltd., Shanghai, China, 2086-83-1). Following a seven-day acclimatization period, the mice were randomly divided into three experimental groups (8 mice per group). The experimental design included the following treatment groups. (1) Control group (Ctrl): received drinking water for 10 days, followed by daily oral administration of PBS for 4 days; (2) DSS group: administered 2.5% DSS in drinking water for 10 days, followed by daily oral PBS for 4 days; (3) DSS + Berberine group: received 2.5% DSS along with oral berberine treatment for 10 days, followed by continued daily oral administration of berberine for an additional 4 days. The mice were euthanized, and the blood, gastrointestinal tissues, feces, and intestinal contents were collected for further analysis. All samples were stored at −80 °C for further analysis.

### 4.3. Intestinal Barrier Function Assays

Colon tissues were analyzed by hematoxylin and eosin (H&E) staining and alcian blue-periodic acid-schiff (AB-PAS) staining according to the method described by Hu et al. [55]. The immunohistochemistry of colon tissues was analyzed using an anti-Muc2 antibody (Invitrogen, Carlsbad, CA, USA). Representative photomicrographs were captured using a Leica DMIL microscope integrated with a DFC450C digital camera (Leica Microsystems, Wetzlar, Germany). Lipocalin-2 (LCN-2) was assayed via an assay kit according to the manufacturer’s protocol (Invitrogen, Carlsbad, CA, USA).

### 4.4. RT–qPCR Analyses

Total RNA was extracted from colon tissues using the RNAiso Plus (Takara, Kyoto, Japan) in accordance with the manufacturer’s protocol for subsequent gene expression analysis [56,57]. RT-qPCR primers for genes of interest (Supplemmentary Table S1) were then used for the relative quantification of the gene expression using TB Green Premix Ex Taq II (Takara, RR820Q) on the StepOne Plus RT-PCR System (Thermo Fisher, Waltham, MA, USA) by using 2^–ΔΔCT^ methodology.

### 4.5. Analysis of IgA and IgA-Coated Bacteria

IgA was determined in frozen fecal pellets via Mouse IgA ELISA Kit according to the manufacturer’s protocol (E-EL-M0690, Elabscience (Wuhan, China)). The experimental procedures for assessing IgA-coated bacteria were performed as previously described by Gabriel et al. [29], with minor modifications. Samples were homogenized in 500 µL ice-cold PBS by mixing at maximum speed on a ThermoMixer (Thermo Scientific, Hamburg, Germany) for 20 min at 4 °C. Samples were topped up with 500 µL PBS, followed by centrifugation at 100× *g* for 5 min at 4 °C. The supernatant was passed through a 70 µm strainer, followed by centrifugation at 10,000× *g* for 5 min at 4 °C. The pellet was resuspended with 1 mL PBS for measurement of the optical density at 600 nm and quantification of the bacteria. Samples were incubated with 500 µL PBS with 5% goat serum (Gibco, 11540526, Fisher Scientific (Waltham, MA, USA)) at 4 °C for 20 min. After centrifugation at 10,000× *g* for 5 min at 4 °C, pellets were resuspended in PBS with 5% goat serum and the appropriate antibody: FITC-conjugated anti-mouse IgA (Clone mA-6E1, eBioscience (Hangzhou, China), 11-4204-83, Life Technologies (Shanghai, China)). After incubating for 30 min at 4 °C, samples were washed in 1 mL PBS for data acquisition on a NovoCyte Quanteon flow cytometer (ACEA Biosciences, San Diego, CA, USA).

### 4.6. 16S rRNA Sequencing of Fecal Bacteria

Bacterial DNA was extracted from colonic content samples using a DNA isolation kit (DP328, TIANGEN (Beijing, China)) following the manufacturer’s protocol. The V3–V4 region was amplified and sequenced. Subsequently, the 16S rRNA sequences of fecal bacteria were then aligned and taxonomically classified using the SILVA reference database. Finally, an ASV table was produced for abundance information and further analysis on MicrobiomeAnalyst 2.0. Alpha diversity was assessed using the Chao1 and Ace indices (to estimate species richness) and the Shannon index (to evaluate both richness and evenness). To specifically infer the metagenomic potential for mucin degradation, we performed functional prediction from the 16S rRNA gene data using PICRUSt2.

### 4.7. Mucinase Activity Analysis

Colonic content mucinase activity was assessed according to previously described methods [58]. To normalize mucinase activity to protein content, total protein concentration in the fecal homogenates was measured using the BCA assay. To further analyze microbiota to utilize mucin to grow, we cultured a fecal sample in a medium with porcine stomach mucin as a carbon source after culturing for 36 h under aerobic and anaerobic conditions at 37 °C.

### 4.8. Quantification of SCFAs in Colonic Contents

SCFAs were measured using the previous method with minor modifications [59]. SCFAs were analyzed by Agilent GC-MS 5975 equipped with a DB-WAX column (30 cm × 0.32 mm × 0.5 μm) (Agilent, Santa Clara, CA, USA). The standard curve was used to quantify SCFAs (acetate, propionate, isobutyric acid, butyrate, isovaleric acid, and valeric acid).

### 4.9. Statistical Analysis

Data are presented as mean ± SEM. Statistical comparisons among groups were performed using one-way ANOVA followed by multiple comparison tests and non-paired *t*-test in GraphPad Prism 10.0 (GraphPad Software, San Diego, CA, USA). Significance levels were denoted as follows: * *p* < 0.05, ** *p <* 0.01, *** *p* < 0.001, and NS means no significance. Adobe Illustrator software (version 2024) was used for drawing.

## 5. Conclusions

Taken together, our results demonstrate that berberine supplementation enhances intestinal barrier integrity in a model of colitis. This effect was strongly correlated with the upregulation of key mucosal defense factors, including IgA, IgA-coated bacteria, Lyz1, Reg3*g*, and Pla2g2*a*, which were in turn linked to a restructured and stabilized gut microbiota. Furthermore, the barrier-protective benefits of berberine were largely attributable to a promoted mucin–SCFA metabolic axis, driven by enriched *Akkermansia* and *Bacteroides*. Collectively, our findings reveal a novel mechanism through which berberine alleviates intestinal inflammation, providing a scientific foundation for its development as a functional feed additive aimed at improving gut health and productivity in livestock.

## Data Availability

The data presented in this study are openly available in NCBI (accession no: PRJNA1119245). Further inquiries can be directed at the corresponding authors.

## Supplementary Materials

The following supporting information can be downloaded at: https://www.mdpi.com/article/10.3390/ijms27031549/s1.

## Abbreviations

The following abbreviations are used in this manuscript:

AB-PAS	Alcian blue-periodic acid-Schiff
DAI	Disease activity index
DSS	Dextran sulfate sodium
ER	Endoplasmic reticulum
H&E	Hematoxylin and eosin
PCR	Polymerase Chain Reaction
IBD	Inflammatory bowel disease
SCFA	Short-chain fatty acids
ANOVA	Analysis of Variance
