# Supplementary material for: Berberine Ameliorates DSS-Induced Colitis via Regulation of Mucosal Barrier Homeostasis and Mucin-Degrading Microbiota
Source: Int J Mol Sci. 2026 Feb 4;27(3):1549. doi: 10.3390/ijms27031549 (PMC12897809; doi:10.3390/ijms27031549)
Supplement: Supplementary file 1 [file ijms-27-01549-s001.zip › ijms-4092078-supplementary-Figure S1.pdf]

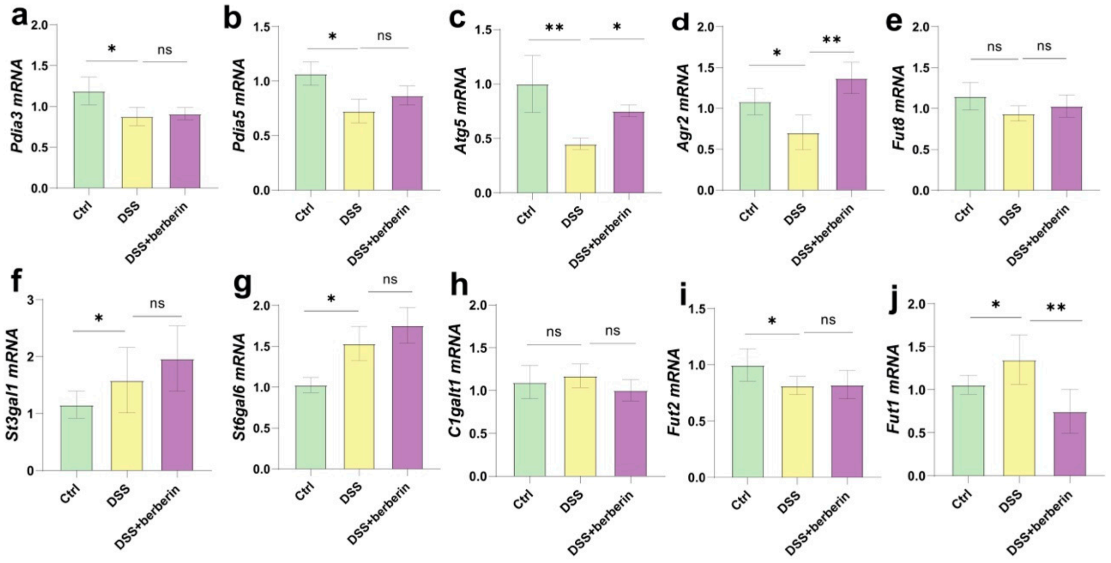

Figure S1. Berberine impact mucus production and secretion. (a-e) Relative mRNA expression levels of markers associated with mucus layer secretion. (a) Protein disulphide isomerase A3 (*Pdia3*), (b) Protein disulphide isomerase A3(*Pdia5*), (c) Autophagy protein 5 (*Atg5*). (d)Anterior gradient 2(*Agr2*). (e-j) mRNA relative expression of glycosyltransferases involved in mucin glycosylation. (e) Fucosyltransferase 8 (*Fut8*), (f) ST3 b-galactoside a-2,3-sialyltransferase 1 (*St3gal*), (g) ST6 N-acetylgalactosaminide a-2,6-sialyltransferase 2 (*St6gal6*), (h) glycoprotein-N-acetylgalactosamine 3-beta-galactosyltransferase 1 (*C1galt1*), (i, j) fucosyltransferase 2/1 (*Fut2*, *Fut1*).
