# Supplementary material for: Berberine Ameliorates DSS-Induced Colitis via Regulation of Mucosal Barrier Homeostasis and Mucin-Degrading Microbiota
Source: Int J Mol Sci. 2026 Feb 4;27(3):1549. doi: 10.3390/ijms27031549 (PMC12897809; doi:10.3390/ijms27031549)
Supplement: Supplementary file 1 [file ijms-27-01549-s001.zip › ijms-4092078-supplementary-Table S1.pdf]

Supplementary Table S1. Primers used for q-PCR.

gene	Primer sequences (5′ to 3′)	
	Forward	Reverse
Hes1	CTGGATAGAAGCCGCGCTC	AGGTCATGGCGTTGATCTGG
Dmbt1	CAAGTCTCCATCACCCAAACAT	CCCCGAGCAAGACACATCAT
Klf4	GTGCCCCGACTAACCGTTG	GTCGTTGAACTCCTCGGTCT
Elf3	CCTATGAGAAGCTGAGCCGA	ACCTCTTCTTCCTTCCAGCC
Atoch	AACAGCGATGATGGCACAGA	GTTGCCTTCCTAACTGGCCT
Spdef	AGGTGCAATCGATGGTTGTG	AGGGTCTGCTGTGATGTTCA
Gsdmc4	TGAGGAGCCTGCCAATCTAAA	ATGTGGGGTGCTAGAATCCTT
CLCA1	AACAACGGCTATGAGGGCATC	TTGATACAAGGACATCAGCGTTT
Muc2	ATGCCCACCTCCTCAAAGAC	GTAGTTTCCGTTGGAACAGTGAA
Agr2	GCCAAAGACACCACAGTCAA	CCATCAAGGGTCTGTTGCTT
TFF3	AGATTACGTTGGCCTGTCTCC	AGATCGGGGATGCTTGCTAC
Fcgbp	TAGATGGGCACCGGTTTGAC	CGAACTCTCCGTTCACCTGT
Nlrp6	CCCGAAATGTCATCTGAGTGTTCT	TTCAGGGCCTCGGAAAGGT
Pdia3	CGCCTCCGATGTGTTGGAA	CAGTGCAATCCACCTTTGCTAA
Retnlb	CAAGGAAGCTCTCAGTCGTCAA	CACTAGTGCAGGAGATCGTCTTAG
Pdia5	GACCCGCAATAACGTGCTG	GACCCGCAATAACGTGCTG
Atg5	ATGGTTTGAATATGAAGGCACACC	TGATGTTCCAAGGAAGAGCTGAA
Fut8	AGGCGAATGGCTGAGTCTCT	TGGCCTTAACAAGCTGTTCTTCT
St3gal1	GCCCACTATGCCAGACACTT	TCAGCAGAGTCAAACCCAGC
St3gal4	GGCTCTGGTCCTTGTTGTTG	TCCCTAGAACGGTTGCCAAAA
St6gal6	CACCCCAAAAGCGCAGATTTATT	CCTGCCTGAAACAGAGTCCAA
Clgalt1	ATGGACACAGTCACCTCAAAGG	GAGGTTCTCAGCAACGTCTATGT
Fut2	TGAACTTTCGGCTAAGGTACATCT	GGAAGTGGGCCAGAGGAAAG
Fut1	AGAATTCGCTTGCACCACCA	AAGAAGGAGCCGGCAGAGA

ZO-1	TGCCATTACACGGTCCTCTG	GGTTCTGCCTCATCATTTCCTC
Occludin	AGTGTGATAATAGTGAGTGCTATCC	TGTCATACCTGTCCATCTTTCTTC
Claudin-1	TTCTCGCCTTCCTGGGATG	CTTGAACGATTCTATTGCCATACC
Zg 16	CTCGGCCTCTGCTAATTCCA	CGCACCTGGAGACCTACTAT
Lyz1	GCCAAGGTCTACAATCGTTGTGAGTTG	CAGTCAGCCAGCTTGACACCACG
Reg3g	ATGCCCCATCTTCACGTAGC	GGGTTCATAGCCCAGTGTCG
Pla2g2a	GACTGTTGCTACAAGAGCCTGG	ATCGCACTGACACAGCCGTTTC
Slfn2	ATGGGTACTAGACTTGAGGCAA	AGCTTAGCATATTTGGCTTCCAG
actin	CATCCGTAAAGACCTCTATGCCAAC	ATGGAGCCACCGATCCACA
Saa1	CCAGGAGACACCAGCAGGAT	GTGTCCTCATGTCCTCTGCC
